# Impact of 980 nm diode laser activated irrigation on adaptability of root canal filling in oval-shaped canals

**DOI:** 10.1038/s41405-025-00354-4

**Published:** 2025-07-17

**Authors:** Sabah M. Sobhy

**Affiliations:** Endodontic Department, Faculty of Dental Medicine for Girls, Al-Azhar University, Cairo, Egypt

**Keywords:** Occupational health, Endodontics

## Abstract

**Aim:**

To assess the impact of 980 nm diode laser-activated irrigation with or without EDTA on the adaptability of root canal filling in oval-shaped canals using a scanning electron microscope.

**Methodology:**

Forty-five single-rooted mandibular premolars were assigned into three groups (*n *= 15) based on the final irrigation protocol: Conventional irrigation without activation; 980 nm diode laser without EDTA; 980 nm diode laser with EDTA. The marginal gap width (μm) and surface area (μm²) between the obturating material and the root canal were measured in the coronal, middle, and apical thirds using SEM software (ImageJ software version 1.53 t). The data was analyzed using Two-way ANOVA and Bonferroni’s post hoc tests.

**Results:**

All groups demonstrated statistically significant differences in mean gap width and surface area (*p *< 0.001) at the coronal, middle and apical root sections. Diode laser with EDTA group had the lowest gap values, followed by the diode laser group, while the conventional group recorded the highest values. Diode laser group exhibited the highest gap value in the coronal section. Additionally, significant differences in mean gap width and surface area were observed at different root levels within each group (*p *< 0.001). The highest mean gap value observed in the apical section, whereas the lowest value was found in the middle section for both the diode laser with EDTA and diode laser groups. In contrast, the conventional group had its lowest value in the coronal section.

**Conclusion:**

Activation of the 2.6% NaOCl and 17% EDTA using 980 nm diode laser improves the adaptation of the root canal filling to the canal walls.

## Introduction

Root canal anatomy is complex and distinguished by accessory structures such as the isthmus, fins, and apical delta, making root canal therapy challenging. A successful root canal therapy requires proper biomechanical preparation, irrigation, and root canal obturation. Irrigation is essential in root canal therapy as it enables cleaning of areas that are inaccessible to mechanical preparation [1]. To effectively removes microorganisms from the complex root canal system, adherence to an appropriate irrigation protocol is essential [2, 3].

Traditionally, root canals have been cleaned and shaped using chemo mechanical preparation which aiming to eliminate infected pulp tissue and dentin while shaping the canals for subsequent obturation. However, about 35% of the root canal surface remains untouched by the instruments particularly in large oval-shaped canals where the presence of untouched buccal and lingual extensions or recesses might harbor bacterial biofilms and tissue debris and irrigants lack the ability to deeply penetrate into these areas [4–6].

For many years, sodium hypochlorite (NaOCl) has been employed as the primary irrigating solution in root canal treatment. It has a significant bactericidal impact upon direct contact in vitro; however, it’s in vivo effect is limited due to its inability to remove the smear layer and penetrate deep into the dentinal tubules where bacteria reside [7]. NaOCl dissolves organic components of dentin while ethylenediaminetetraacetic acid (EDTA) efficiently removes the inorganic components [8]. Therefore, combining 2.5% NaOCl and 17% EDTA during root canal therapy is recommended for optimal smear layer removal [9].

Many delivery devices and techniques have been developed to enhance the effectiveness of irrigating solutions by improving their flow and dispersion within the root canal. Brushes, manual files, gutta-percha cones, ultrasonic, sonic, and laser devices are among the tools used [10]. As laser techniques and devices have advanced, diode laser has grown in popularity due to the small size and low cost. It is ideal for root canal therapy due to its infrared wavelength and the ability to employ thin, flexible fibers [6].

The available wavelengths for dental application vary between 800 and 1064 nm. Diode laser has broad range of indications owing to their strong absorption in pigments such as hemoglobin and melanin as well as their ability to transmit through water and hydroxyapatite. It promotes cell proliferation while inhibiting inflammation related enzymes. Diode laser is expected to achieve deep penetration in dentin (up to 1000 μm) and demonstrate selective bactericidal effects. A study showed that 980 nm diode laser has bactericidal effect ranging between 77% and 97% in root canals infected with Enterococcus faecalis when energy outputs of 1.7, 2.3 and 2.8 W were used. Antimicrobial effect was found to be influenced by both the energy level and dentin thickness [11].

Diode lasers have garnered significant attention from researchers in recent years due to their bactericidal properties through thermal effects. Studies have shown varying levels of success in achieving root canal disinfection using diode lasers [12–17]. When used with care, diode lasers can serve as a valuable tool, simplifying various dental procedures, saving time [18] and enhancing the quality of dental patients care [19, 20]. Laser-activated irrigation has become a topic of interest in endodontics, as it enhances irrigant warming while simultaneously agitating it, thereby increasing its effectiveness. Combining a diode laser with EDTA solution has been reported as an effective approach for smear layer elimination [21–23]. This process exposes the dentinal tubules, enhancing the penetration of irrigants. Consequently, introducing the sealer into these open tubules during obturation provides a better seal for the canal [24]. Diode laser can be a valuable addition to the armamentarium used for smear layer removal along with its antimicrobial properties against root canal microbes may enhance the success of endodontic treatment [25]. Kaur et al. compared the effectiveness of ultrasonic and diode laser irrigation activation techniques in removing the smear layer from the root canal dentin and the results revealed that diode laser activation showed better cleaning of root dentinal walls compared to ultrasonic activation [26].

The marginal adaptability of root canal filling material is critical for maintaining long term stability of treatment outcomes. The main aim of endodontic obturation is to produce a hermetic fluid seal. It is the final phase in endodontic therapy that keeps inaccessible bacteria and their byproducts inside the canal while also preventing the transfer of fluids and nutrients into it. Since the majority of treatment failures were due to inadequate obturation, insufficiently filled gaps may serve as a breeding ground for bacteria [27]. Therefore, this study aimed to evaluate the effect of 980 nm diode laser activated irrigation with or without EDTA on adaptability of root canal filling in oval-shaped canals utilizing a scanning electron microscope.

The null hypothesis stated that there is no difference in adaptability of root canal filling when using 980 nm diode laser-activated irrigation (with or without EDTA) in oval-shaped canals compared to conventional irrigation technique.

## Materials and method

### Study design

**Fig. 1:**
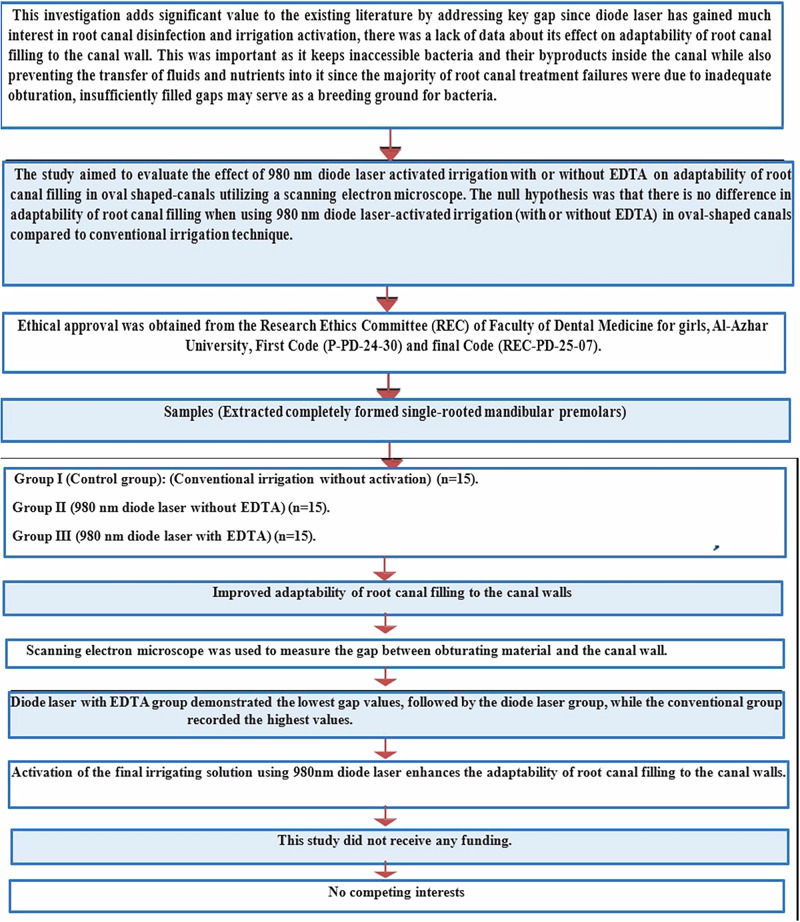
PRILE 2021 flowchart of 980 nm diode laser activated irrigation effect on adaptability of root canal filling.

This randomized in vitro study was conducted in the Endodontic Department at the Faculty of Dental Medicine for Girls, Al-Azhar University. It was designed, reported and written according to PRILE (Preferred Reporting Items for Laboratory studies in Endodontology) 2021 guidelines Fig. 1 [28]. Ethical approval for the use of extracted human teeth was obtained from the Research Ethics Committee (REC) of Faculty of Dental Medicine for girls, Al-Azhar University, Cairo – Egypt, primary Code (P-PD-24-30) and final Code (REC-PD-25-07).

### Sample selection

Human permanent completely formed single-rooted mandibular premolars which were recently extracted for periodontal or orthodontic causes were selected in this study. Periapical radiographs (buccal and proximal views) were taken for each tooth to verify the presence of a single, oval-shaped canal.

#### Inclusion criteria

1. Human single rooted mandibular premolars.
2. Single straight canal.
3. Mature apices.
4. Roots without signs of cracks or caries or fracture.
5. No evidence of root canal calcification or internal resorption.
6. Teeth have no previous root canal treatment.

#### Exclusion criteria

1. Teeth with more than one root canal.
2. Teeth with evidence of root caries, cracks or fractures.
3. Teeth with previous root canal treatment.
4. Teeth with any abnormalities as internal, external resorption or root canal calcification.
5. Teeth with open root apex.

### Sample size calculation

The sample size calculation was conducted utilizing G*Power version 3.1.9.7 based on data of a previous study [6]. A power analysis was designed to ensure sufficient statistical power for a two-sided test to reject the null hypothesis which assumes no differences between groups. The calculation was performed with an alpha level of (0.05) and a beta of (0.05), i.e. power = 95% and an effect size (d) of (0.62) calculated based on the previous study’s results. The predicted sample size (*n*) was 45, i.e., 15 samples per group. To identify differences across coronal, middle and apical regions between groups.

### Sample preparation and grouping

The samples were decoronated utilizing diamond discs (Diatech, GoltèneAG, Switzerland) under copious water cooling to standardize the root length to 15 ± 1 mm for all samples. Root canal preparation was carried out with EdgeFile X7 nickel-titanium rotary system (Edge Endo, Albuquerque, New Mexico, USA) driven by EndoEst motor mini (Geosoft Dent., Russia) endomotor till #40 taper 0.04 file in continuous rotation at 300 rpm with a torque of 2 Ncm. After each file usage, the root canal irrigation was performed using 2 ml of freshly prepared 2.6% sodium hypochlorite (NaOCl) solution (Alex. Deteregents and Chemical Co., Egypt) for 1 minute with a 31-gauge Navi-Tip flexible irrigating needle (Navi-Tip, Ultradent product, South Jourdan, UT) [27].

The samples were randomly assigned to groups based on the final irrigation protocol using simple randomization procedure using random number generator (https://www.random.org/) into three equal groups (*N *= 15). Allocation concealment was carried out by a dental student, who placed each sample into an envelope, thoroughly shuffled the envelopes, and sequentially numbered them from 1 to 45:

#### Group I (Control group): (Conventional irrigation without activation)

15 roots were irrigated with 5 mL of 2.6% NaOCl (Alex. Deteregents and Chemical Co., Egypt) for 2 min followed by 5 mL 17% EDTA (Colgate Oral Care Company, Waverly, Australia) for 1 minute with 5 mL distilled water in between and as final rinse. The root canals were subsequently dried using paper points.

#### Group II (980 nm diode laser without EDTA)

15 roots were irrigated with 5 mL of 2.6% NaOCl that were activated by the 980 nm diode laser with 200 μm fiber optic tip (Lite medics, Italy) for a total of 20 seconds in pulsed mode and 1.2watt power. Each root was subjected to 4 lasing cycles each lasting 5 seconds with 20 seconds intervals in between cycles. For each root, the lasing cycle was applied four times, using 1.25 ml of 2.6% NaOCL each time. The fiber tip was positioned 1 mm short of the working length, activated and moved in slow helical motion from the apex to the cervical third, alternating between clockwise and counterclockwise direction at speed of approximately 2 mm/s. This was done to ensure equal distribution of light inside the root canal [25]. Then, the canals were rinsed with distilled water (5 mL) and dried.

#### Group III (980 nm diode laser with EDTA)

15 roots were irrigated with the same technique that was applied in group II. However; after canal rinsing with 5 mL distilled water, same irradiation protocol was applied with 5 ml 17% EDTA. 1.25 mL of EDTA was used at each lasing cycle [6]. The canals were then, rinsed with distilled water (5 mL) and dried.

**Fig. 2:**
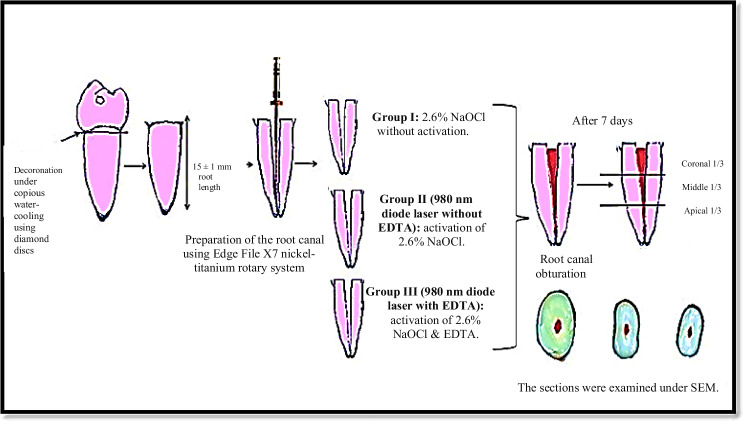
Schematic presentation of specimen preparation and grouping.

The root canal system was obturated using size 40 taper 0.04 gutta-percha cone (Dentsply-Maillefer, Ballaigues, Switzerland) and resin-based sealer, ADSEAL (Meta Biomed Co, Cheongju, Korea) through cold lateral compaction method after canal dryness using paper points that matched the size of the master cone. Periapical radiographs (proximal and buccal views) were taken to check obturation quality and glass ionomer filling material was then be used to seal the root canal orifices. The specimens were then incubated at a 37 ^o^C with 95% relative humidity for 7 days to allow complete setting of sealers [29]. A schematic diagram illustrating the specimen preparation is presented in Fig. 2.

### Scanning electron microscope evaluation

After 7 days, root sections were cut transversely at 3, 8 and 13 mm from the root apex to represent the center of coronal, middle and apical thirds of the root using diamond disk (Diatech, GoltèneAG, Switzerland). Each section was reduced to a thickness of 1 mm, rinsed with 17% EDTA for 15 seconds, followed by a rinse with distilled water for an equal duration to eliminate the smear layer and then stored in a sealed container. The sections were affixed on an aluminum stub using carbon double adhesive tape and observed under a scanning electron microscope (Prisma E SEM /FEI Quanta 3D 200i Edx/ Termo Fisher Scientific-US) at an accelerating voltage of 20 kV under X2000 magnification.

The marginal gap width (μm) and surface area (μm^2^) between the canal walls and the obturating material at three randomly chosen points in each section of the coronal, middle and apical root thirds were measured using SEM software (ImageJ software version 1.53 t). The average value for each specimen was then determined and the procedure was repeated for each section of each tooth to evaluate the adaptability of root canal filling to the canal wall [29, 30].

$${{{\rm{Gap}}}}\; {{{\rm{Surface}}}}\; {{{\rm{Area}}}}\left({{{{\rm{\mu }}}}{{{\rm{m}}}}}^{2}\right)={{{\rm{Gap}}}}\; {{{\rm{Length}}}}\left({{{\rm{\mu }}}}{{{\rm{m}}}}\right)\times {{{\rm{Average}}}}\; {{{\rm{Gap}}}}\; {{{\rm{Width}}}}({{{\rm{\mu }}}}{{{\rm{m}}}}).$$

### Statistical analysis

Statistical analysis was performed using SPSS software version 20 (Statistical Package for Scientific Studies, SPSS, Inc., Chicago, IL, USA) for Windows. Numerical data were presented as mean and standard deviation. Data normality was assessed by examining the distribution using Kolmogorov–Smirnov and Shapiro–Wilk tests. Since the data followed a parametric distribution, comparisons of means between and within groups were performed using the Two-way ANOVA test, followed by Bonferroni’s post hoc test for pairwise comparisons.

All *p* values were two-sided. *P* values ≤ 0.05 were considered significant.

## Results

### Gap width (μm)

**Fig. 3:**
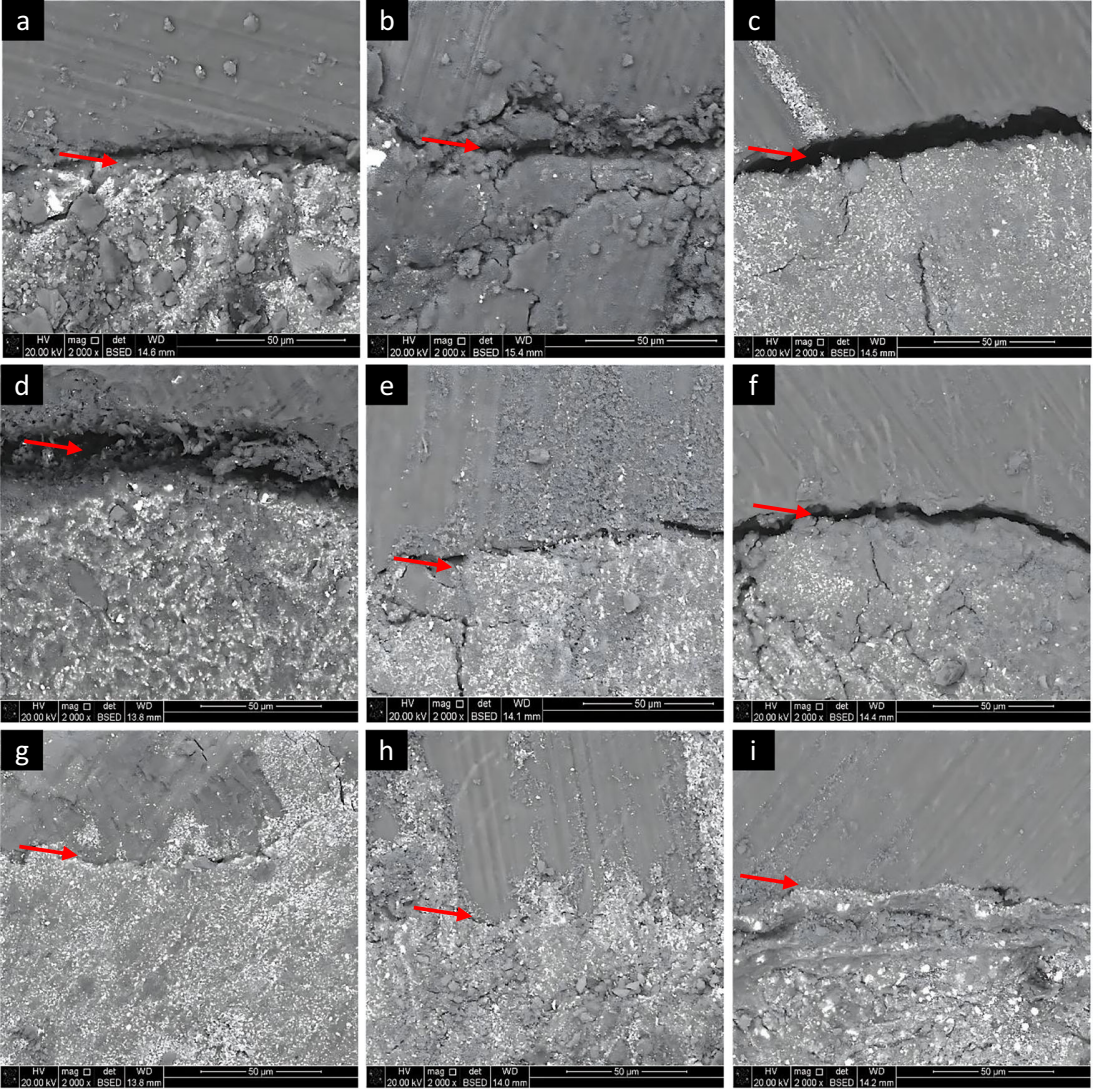
SEM images demonstrating the interfacial gap between root canal wall and the obturating material at different root levels. **a**–**c** Conventional irrigation without activation group in the coronal (**a**), middle (**b**) and (**c**) apical sections. **d**–**f** 980 nm diode laser without EDTA in the coronal (**d**), middle (**e**) and (**f**) apical sections. **g**–**i** in the coronal (**g**), middle (**h**) and (**i**) apical sections.

**Fig. 4:**
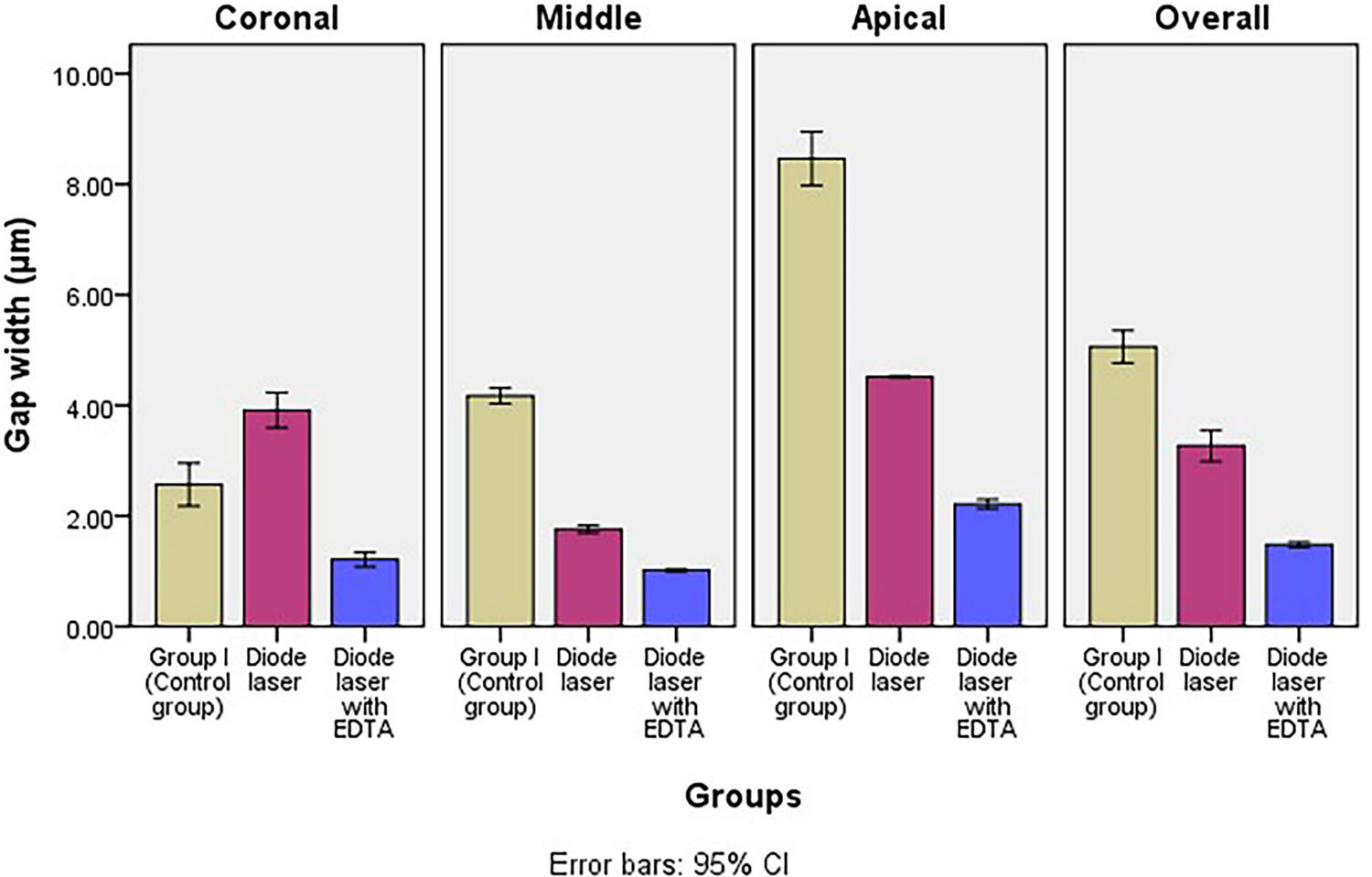
Bar chart illustrating mean gap width (μm) in different groups.

Two-way ANOVA test revealed a statistically significant difference in mean gap width among the groups across the coronal, middle, and apical sections (*p *< 0.001). Bonferroni’s post hoc tests showed that Diode laser with EDTA group exhibited the lowest mean gap width (1.48cB ± 0.07), followed by Diode laser group (3.27bC ± 0.40), while the conventional group had the highest mean gap width (5.06aB ± 0.41) (Table 1 and Figs. 3 and 4).

**Table 1:** Comparison between gap width (μm) of different groups and between different sections within each group (two-way ANOVA test).

Group	Coronal third	Middle third	Apical third	Overall	*P* value (within group)
Group I (Control group)	2.57bD ± 0.54	4.17aC ± 0.20	8.46 aA ± 0.68	5.06aB ± 0.41	0.000^*^
Group II: Diode laser	3.91aB ± 0.45	1.76bD ± 0.10	4.52bA ± 0.02	3.27bC ± 0.40	0.000^*^
Group III: Diode laser with EDTA	1.21cC ± 0.18	1.02cD ± 0.03	2.21cA ± 0.12	1.48cB ± 0.07	0.000^*^
*P* value (between groups)	0.000^*^	0.000^*^	0.000^*^	0.000^*^	

Significance level *p *≤ 0.05, ^*^Significant

Within each group, Two-way ANOVA test revealed a statistically significant difference in mean gap width values between the coronal, middle, and apical sections (*p *< 0.001). In all groups, the apical section exhibited the highest mean gap width value. The lowest gap width was recorded in the middle section for both Diode laser with EDTA and Diode laser groups, whereas in the conventional group, the lowest value was found in the coronal section. Diode laser group had the highest mean gap width in the coronal section (3.91aB ± 0.45).

### Gap surface area (μm2)

**Fig. 5:**
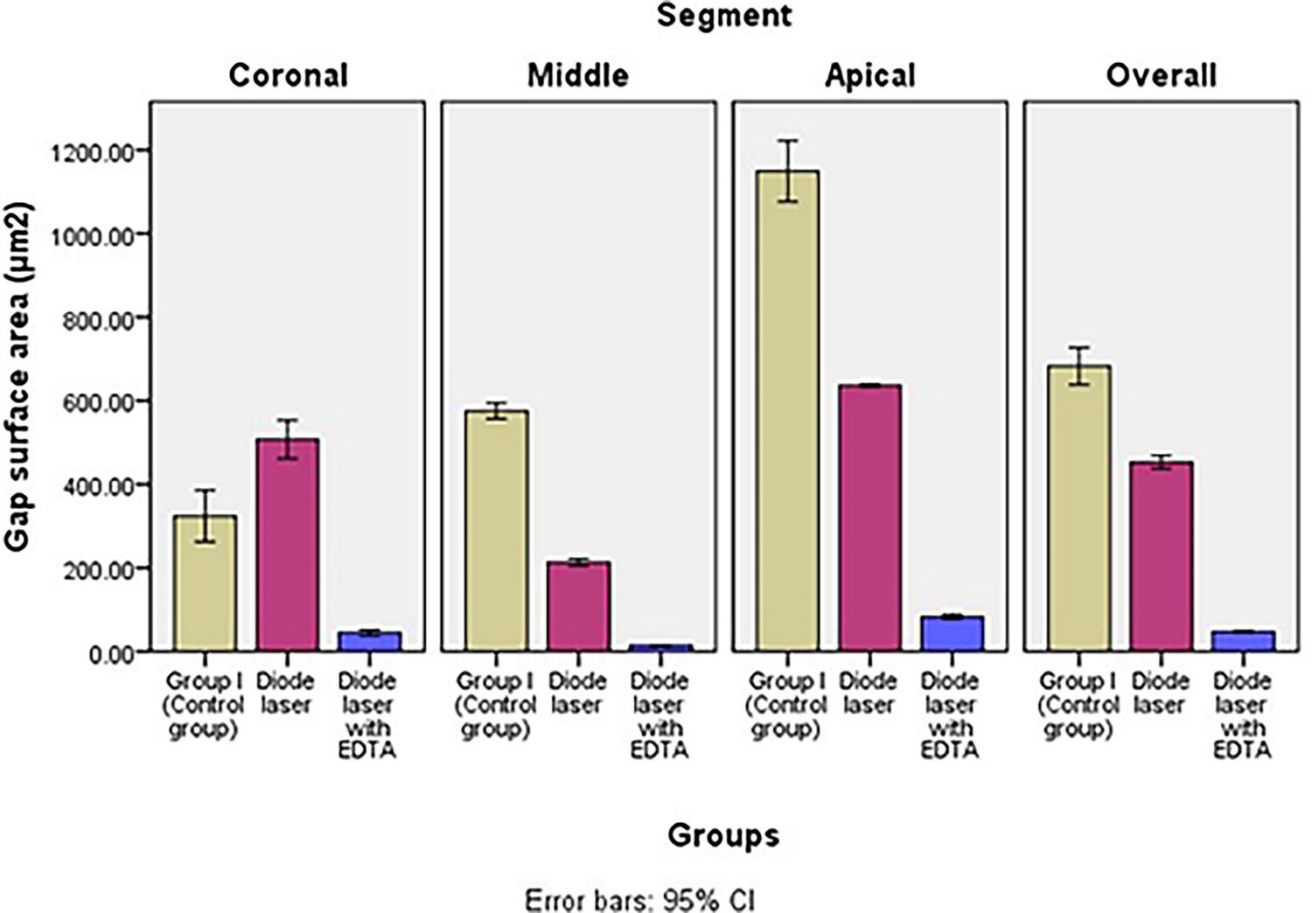
Bar chart illustrating mean gap surface area (μm^2^) in different groups.

Table 2, Fig. 5. Two-way ANOVA test revealed a statistically significant difference in mean gap surface area among the groups across the coronal, middle, and apical sections (*p *< 0.001). Bonferroni’s post hoc tests showed that Diode laser with EDTA group exhibited the lowest mean gap surface area (47.31cB ± 2.79), followed by Diode laser group (452.34bC ± 21.89), while the conventional group had the highest mean gap width (682.82aB ± 62.40).

**Table 2:** Comparison between gap surface area (μm^2^) of different groups and between different sections within each group (ANOVA test).

Group	Coronal third	Middle third	Apical third	Overall	*P* value (within group)
Group I (Control group)	323.63bD ± 86.26	575.47aC ± 26.65	1149.42 aA±102.00	682.82aB ± 62.40	0.000^*^
Group II: Diode laser	507.44aB ± 64.20	213.12bD ± 11.58	636.58bA ± 5.42	452.34bC ± 21.89	0.000^*^
Group III: Diode laser with EDTA	44.68cB ± 9.44	14.62cC ± 0.66	82.65cA ± 8.50	47.31cB ± 2.79	0.000^*^
*P* value (between groups)	0.000^*^	0.000^*^	0.000^*^	0.000^*^	

Significance level *p *≤ 0.05, ^*^significant.

Within each group, Two-way ANOVA test revealed a statistically significant difference in mean gap surface area values between the coronal, middle, and apical sections (*p *< 0.001). In all groups, the apical section exhibited the highest mean gap surface area. The lowest gap surface area was recorded in the middle section for both Diode laser with EDTA and Diode laser groups, whereas in the conventional group, the lowest value was found in the coronal section. Diode laser group had the highest mean gap surface area in the coronal section (507.44aB ± 64.20).

## Discussion

Achieving successful root canal treatment necessitates adequate biomechanical preparation, effective irrigation and proper obturation. Irrigation has a vital role in root canal treatment, as it enhances the cleaning of areas that mechanical preparation alone cannot reach [2, 3]. The main objective of endodontic obturation is to establish a hermetic fluid seal and prevent inaccessible bacteria and their byproducts from remaining within the canal while also blocking the movement of fluids and nutrients [27]. While several studies have explored 980 nm diode laser activated irrigation on smear layer removal and dentinal tubule exposure, to our knowledge, few have specifically investigated its effect on the adaptation of root canal fillings in oval-shaped canals. The adaptability values of all the groups were statistically significantly different (*p *< 0.001) through all root sections (coronal, middle, and apical) thus, the null hypothesis of this study was rejected.

The combination of NaOCl and EDTA is the most commonly used regimen for elimination of smear layer in the root canal system, creating rough dentin surfaces that improve micromechanical bonding to the canal walls [31]. However, EDTA activation promotes improved irrigant penetration, effectively cleans canal walls, removes the smear layer and opens dentinal tubules across all sections of the root canal [6]. Therefore, this study aimed to evaluate the effect of 980 nm diode laser-activated irrigation, with or without EDTA, on the adaptability of root canal filling in oval-shaped canals using a scanning electron microscope.

Oval-shaped canals were utilized in this study because they present a greater challenge in achieving complete debridement, disinfection, and subsequent obturation. Their irregular shape often leads to areas that are difficult to clean and fill, increasing the risk of inadequate obturation [32, 33]. Assessing the efficacy of various irrigation activation techniques in these canals provides valuable insights into optimizing endodontic treatment outcomes.

This study found a statistically significant difference in the mean gap width and surface area between different groups in the coronal, middle, and apical sections of the root (*p *< 0.001), diode laser activation of EDTA produced the lowest gap values, meaning it led to the most effective adaptation of the filling material to the canal walls. This suggests that diode laser activation of EDTA enhances the removal of smear layer and improves dentin permeability. Consequently, this could contribute to better sealing, reduced microleakage and potentially improved long term endodontic success. The combined use of a diode laser with EDTA, demonstrated superior smear layer elimination compared to using EDTA, and diode laser alone particularly in the apical region [25]. Elkhodary et al. [6] revealed that 980 nm diode laser activated EDTA irrigation effectively removed the smear layer, opened dentinal tubules without inducing any morphological alterations in the root canal dentin.

Diode lasers provide deeper penetration, reaching up to 500 μm, compared to irrigating solutions, which penetrate only 100 μm and leads to vaporization of the smear layer, thereby enhancing the effectiveness of EDTA [34, 35]. It causes dentin dehydration which enhances the sealing ability of a hydrophobic epoxy resin sealer(adseal) [36]. It has been noted that high-power lasers, when used in combination with NaOCl and EDTA, affect the inorganic / organic ratio of root dentin, that could be a contributive factor for retention of the sealers to dentin surface [37, 38].Supporting these findings, earlier studies have also noted the beneficial impact of various laser systems on the adhesion of resin-based sealers to root canal dentin [39, 40]. On the contrary, it has been shown that laser caused partial to complete occlusion of the dentinal tubules [41]. This effect may be attributed to differences in wavelength and variations in the laser application protocol.

The findings of diode laser (activation of NaOCl) without EDTA showed a smaller gap value in the middle and apical thirds in comparison to the conventional irrigation technique (2.6% NaOCl followed by 17% EDTA), while the gap value was higher in the coronal third. This could be attributed to the laser’s ability to enhance the penetration of NaOCl through cavitation and acoustic streaming and effectively removing organic debris. The laser energy likely increased the depth of NaOCl infiltration, leading to better cleaning and subsequently improved adaptation. In contrast, the coronal third exhibited a higher gap value compared to conventional irrigation. This could be due to the absence of EDTA, which plays a critical role in smear layer removal by demineralizing the inorganic component of dentin. In the coronal region, where mechanical instrumentation generates a thick smear layer, the lack of EDTA might have resulted in inadequate tubule opening and leading to a less effective seal. Diode laser alone was more effective than EDTA in eliminating the smear layer in the apical regions [25] as 200μm fiber optic tip allowed for deeper penetration into the apical third of the root canals, enhancing its efficacy [42].

This study demonstrated a statistically significant difference in gap values at different root regions within each group, with the apical section exhibiting the highest mean gap value. This could be attributed to the narrow and curved anatomy of the apical third, which makes complete smear layer removal more challenging and may hinder root canal filling adaptation. Additionally, the lower number of dentinal tubules, the high mineral content and presence of sclerotic dentin in the apical part can reduce the effectiveness of root canal cleaning. This was in accordance with the findings of a previous study which reported the highest smear layer scores in the apical third for both 980 nm diode laser and conventional groups [6]. Moreover, the apical region exhibited the highest mineral content compared to other sections following the use of irrigating solutions combined with different lasers on intraradicular dentin [37]. This increased mineralization likely reduced the bonding of epoxy resin-based sealers in the apical region [43].

The current findings showed that using a 980 nm diode laser for EDTA activation was effective in enhancing root canal filling adaptation to the canal walls. However, because this investigation was conducted in vitro, the complicated oral circumstances, including fluid dynamics, may not have been completely replicated. Also, the present study does not provide a continuous assessment of the full circumferential interface and may miss localized variations or larger gap areas. Additional studies should evaluate root canal filling adaptation to the canal walls using 360° circumferential SEM mapping.

## Conclusion

Within the limitation of the present study, the following conclusion can be drawn:

Activating the final irrigating solution with 980 nm diode laser has been shown to enhance the adaptability of root canal filling to the canal walls. It can be a valuable addition to irrigation techniques, enhancing the success rate of endodontic treatment.

Further studies should be conducted incorporating ultrasonic activation with and without EDTA and using micro-CT for a comprehensive three-dimensional evaluation of gap formation and adaptation. Additionally, future research should explore the effect of different sealer types to better understand their interaction with laser-assisted irrigation techniques.

## Data Availability

The data is available from the corresponding author on request.
